# Evaluation of Atherosclerotic Changes in the Superior Mesenteric Artery Using MDCT

**DOI:** 10.1155/rrp/8711250

**Published:** 2026-09-18

**Authors:** Vehbi Husam, Serhat Kaya

**Affiliations:** ^1^ Department of Radiology, Başakşehir Çam and Sakura City Hospital, Istanbul, Turkey; ^2^ University of Health Sciences, Başakşehir Çam Çam and Sakura City Hospital, Istanbul, Turkey, akdeniz.edu.tr

**Keywords:** atherosclerosis, calcified plaques, cardiovascular disease (CVD), mesenteric ischemia, multidetector computed tomography (MDCT), superior mesenteric artery (SMA)

## Abstract

**Purpose:**

Atherosclerosis is a systemic vascular disease that can affect visceral arteries, including the superior mesenteric artery (SMA), potentially leading to mesenteric ischemia with high morbidity and mortality rates. Despite its clinical importance, SMA involvement remains under‐reported. This study aimed to evaluate the prevalence, morphological characteristics, anatomical distribution, and associated risk factors of SMA atherosclerotic plaques using multidetector computed tomography (MDCT).

**Materials and Methods:**

This retrospective study was conducted using data collected from a University Hospital in Turkey. This study focused on patients referred to the Department of Radiology and examined imaging data obtained between January 2008 and December 2010. Information was gathered by examining MDCT scans, patient reports, laboratory findings, and medical records of 15,000 patients admitted to our University Hospital over a designated period. The anatomical distribution of the aneurysms within the SMA (proximal, middle, distal, or diffuse) was recorded. The presence of concomitant atherosclerosis in other visceral arteries and cardiovascular risk factors, including hypertension (HT), diabetes mellitus (DM), coronary artery disease (CAD), and abnormal lipid profiles, was evaluated. Statistical analysis was performed using SPSS (Version 10.0), and associations were assessed using Pearson’s chi‐square test, with a significance level set at *p* < 0.05.

**Results:**

The prevalence of SMA atherosclerosis was 0.86% in the studied population. Clinical data were available for 102 patients in this study. HT, DM, and CAD were present in 73.5%, 49%, and 44.1% of the patients, respectively. Calcified plaques were the most common type (55.8%), followed by mixed (28.7%) and soft plaques (15.5%).Plaques were predominantly located in the proximal (29.4%) and proximal–middle (22.4%) segments of the SMA, followed by the middle (14.7%), middle–distal (14.7%), distal (4.6%), and diffuse segments (13.9%). All patients demonstrated concomitant abdominal aortic atherosclerosis. Atherosclerotic involvement of other visceral arteries was frequently observed in this study. The renal arteries were the most commonly affected, with bilateral involvement in 49.2% of the patients and unilateral calcified plaques in 23.4% of the patients. Celiac trunk atherosclerosis was detected in 41.8% of patients, splenic artery involvement in 12.4%, and inferior mesenteric artery (IMA) involvement in 27.1%, making it the least affected artery. Lipid profile analysis revealed elevated total cholesterol levels in 38% of the patients and low HDL levels in 25% of the patients although no significant correlation was found between lipid levels and plaque characteristics or distribution.

**Conclusion:**

Contrast‐enhanced MDCT is a reliable, noninvasive imaging modality for detecting and characterizing SMA atherosclerosis, allowing for an accurate assessment of plaque morphology, distribution, and associated vascular involvement. Although relatively uncommon, SMA atherosclerosis is strongly associated with systemic atherosclerotic disease and major cardiovascular risk factors. Routine evaluation of mesenteric vessels during abdominal CT examinations may facilitate early diagnosis and improve clinical outcomes by enabling timely management of patients at risk for mesenteric ischemia.

## 1. Introduction

Atherosclerosis is a chronic, systemic disease affecting the arterial walls, characterized by the accumulation of lipids, inflammatory cells, and fibrous elements, leading to plaque formation. This build‐up causes hardening and narrowing of the arteries, potentially restricting normal blood flow and resulting in significant clinical manifestations depending on the affected arterial territory [1]. It is a leading contributor to cardiovascular diseases (CVDs), such as coronary artery disease (CAD), stroke, and peripheral artery disease (PAD) [2]. Atherosclerosis of the superior mesenteric artery (SMA) is linked to postprandial upper intestinal ischemia and abdominal discomfort in elderly individuals with an increased risk of CVD [3].

Globally, atherosclerosis remains one of the predominant factors of morbidity and mortality [4]. In 2017, CVDs resulted in over 17 million fatalities, with ischemic heart disease and stroke contributing to 85% of the total age‐standardized mortality rate [5]. The prevalence of atherosclerosis continues to rise globally due to aging populations, lifestyle changes, and the increasing burden of multiple risk factors such as hypertension (HT), diabetes mellitus (DM), dyslipidemia, hyperglycemia, and smoking [6]. Additionally, the number of individuals with DM has risen dramatically, with approximately 463 million individuals affected worldwide. This increase in DM incidence worsens atherosclerosis‐related inflammation, thereby doubling the risk of myocardial infarction or stroke among patients with DM.

Turkey has witnessed an increasing prevalence of noncommunicable diseases, such as obesity and DM, driven by factors associated with urbanization, sedentary lifestyles, and poor dietary habits [7]. In 2016, an estimated 3.4 million adults in Turkey were diagnosed with CVD, and this prevalence is expected to rise to 5.4 million by 2035 [8]. In Turkey, patients with CAD exhibited elevated body mass index (BMI), HT, and Type‐2 diabetes compared with individuals in the healthy control group [9]. The incidence of cardiovascular conditions among elderly Turkish patients was as follows: 71.3% for HT, 24.6% for DM, 44.7% for CAD, 35.9% for atrial fibrillation, and 15.5% for renal insufficiency [10].

Despite the global significance of atherosclerosis, there is limited research focusing on atherosclerotic changes in visceral arteries, particularly the SMA, which supplies blood to the intestines. The SMA is especially vulnerable to atherosclerotic changes in elderly individuals and those with risk factors such as HT, DM, and CAD. Although the prevalence of SMA atherosclerosis is lower than that of coronary or carotid artery involvement, it can lead to chronic or acute mesenteric ischemia, both of which are difficult to diagnose and often result in severe clinical consequences if untreated. Timely diagnosis and surgical intervention are essential for lowering the high mortality rate of acute mesenteric ischemia, as early revascularization has been shown to reduce overall mortality by up to 50% [11]. Patients with chronic mesenteric ischemia often experience abdominal pain after eating, a fear of food, and unintended weight loss, with diagnosis frequently delayed due to confusion with other gastrointestinal conditions [12].

Multidetector computed tomography (MDCT) has transformed the diagnosis of SMA atherosclerosis by providing high‐resolution, noninvasive imaging of the arterial walls and lumen, enabling clinicians to detect, characterize, and quantify atherosclerotic plaques [13]. This technique is significant for guiding treatment decisions, particularly in symptomatic patients at risk of developing mesenteric ischemia. The primary objective of this study was to evaluate atherosclerotic changes in the SMA using MDCT in a large cohort of 15,000 patients. We retrospectively analyzed abdominal MDCT scans to identify and characterize atherosclerotic plaques in 129 adult patients, focusing on their prevalence, risk factors (including HT, DM, and CAD), and plaque types (calcified, mixed, and soft plaques). By assessing these factors, we aim to enhance the understanding of SMA atherosclerosis and its clinical implications, ultimately guiding the development of more effective diagnostic and treatment strategies.

The significance of this study lies in its comprehensive evaluation of atherosclerotic changes in the SMA using MDCT in a substantial patient cohort. By analyzing 15,000 MDCT scans and identifying 129 cases of SMA atherosclerosis, this study provides valuable insights into the prevalence, risk factors, and morphological characteristics of atherosclerotic plaques in the SMA. This study not only contributes to the understanding of SMA atherosclerosis, highlighting its association with significant clinical outcomes, such as mesenteric ischemia, but also demonstrates the efficacy of MDCT in guiding timely and accurate diagnosis. This has the potential to improve treatment strategies and reduce the severe consequences associated with untreated SMA atherosclerosis.

## 2. Methodology

### 2.1. Data Collection

This retrospective study gathered data from a tertiary university hospital located in Turkey. The research focused on patients who were referred to the Radiology Department from January 2008 to December 2010. Clinical and imaging data were retrospectively compiled by reviewing MDCT scans, radiological reports, laboratory results, and electronic medical records of 15,000 patients admitted during this timeframe. All CT scans were conducted using dual‐source 64‐ and 128‐slice MDCT machines after administering intravenous contrast. For each scan, 120 mL of nonionic contrast medium was injected intravenously at a rate of 5 mL/s through a 16–18‐gauge antecubital venous cannula. Images were captured during breath holding with a slice thickness of 5 mm and a pitch value between 1.0 and 1.5. Axial images were reconstructed at a 5‐mm slice thickness for standard evaluation. For patients needing detailed vascular analysis, additional thin‐section reconstructions ranging from 0.6 to 1.25 mm were performed. Multiplanar reformatted (MPR), maximum intensity projection (MIP), and computed tomography angiography (CTA) reconstructions were employed to enhance the visualization of the SMA lumen, plaque morphology, and related vascular involvement. Plaque characterization was conducted using both visual assessment and attenuation‐based analysis on CT images. Calcified plaques were identified as high‐attenuation lesions, while soft and mixed plaques were categorized based on their attenuation properties and morphological appearance.

### 2.2. Sample Size

From the 15,000 abdominal CT scans examined, the study included 129 adult patients who had radiologically confirmed SMA atherosclerosis. These patients were aged between 49 and 78 years, with an average age of 72. The group had a nearly equal gender distribution, comprising 64 males and 65 females.

### 2.3. Inclusion Criteria

Patients aged 49–78 years who had undergone abdominal MDCT scans at our university hospital and were found to have atherosclerotic plaques in the SMA were included in the study.

### 2.4. Exclusion Criteria

Patients were excluded if their CT scans did not clearly exhibit SMA plaques or if they had incomplete medical records. Additionally, individuals aged < 49 or > 78 years and those without identifiable atherosclerotic plaques in the SMA were excluded from the study.

### 2.5. Ethical Considerations

The study was conducted in accordance with ethical standards, and all patient data were anonymized to ensure confidentiality. Approval from the Institutional Review Board (IRB) was obtained prior to the commencement of the study.

### 2.6. Statistical Analysis

Statistical analyses were performed using SPSS Windows Version 10.0 (SPSS Inc., Chicago, Illinois, USA). Descriptive statistics were used to summarize the patient demographics and plaque characteristics. Pearson’s chi‐square test was used to examine the relationship between cardiovascular risk factors, such as HT, DM, and CAD, and both the morphology of plaques (categorized as calcified, mixed, or soft) and their anatomical distribution within the SMA. The significance level was set at *p* < 0.05.

### 2.7. Results

A retrospective evaluation was conducted on 15,000 abdominal CT scans of patients admitted to our University Hospital. Among the 129 patients diagnosed with SMA atherosclerosis via radiological methods, comprehensive clinical and laboratory data on risk factors were available for 102 patients. Consequently, statistical analysis of cardiovascular risk factors was conducted in this subgroup. The cohort comprised 129 adult patients with SMA plaques, aged between 49 and 78 years, with a mean age of 72. Of these patients, 64 were male and 65 were female (Table 1). The incidence of SMA atherosclerosis in this population was 0.86%. The median age of the patients was 72 years, with females having a median age of 72 years and males a median age of 70 years.

**TABLE 1:** Demographic characteristics and distribution of superior mesenteric artery (SMA) plaque types and locations.

Variable	*n*	%
*Gender*		
Female	65	50.4
Male	64	49.6

*Plaque type*		
Calcified	72	55.8
Mixed	37	28.7
Soft	20	15.5

*Plaque location*		
Proximal	38	29.4
Middle	19	14.7
Distal	6	4.6
Proximal–middle	29	22.4
Middle–distal	19	14.7
Diffuse involvement	18	13.9

*Note:* Data are presented as numbers (*n*) and percentages (%).

A review of the patient records revealed that 102 of the 129 patients had available records. The prevalence rates of HT, DM, and CAD were 73.5%, 49%, and 44.1%, respectively. In the SMA, 55% of the detected plaques were calcific (72 patients, 39 men and 33 women), 28.7% were mixed (37 patients, 20 women and 17 men), and 15.5% (20 patients, 9 women and 11 men) were of the soft type, as shown in (Table 1).

**FIGURE 1:**
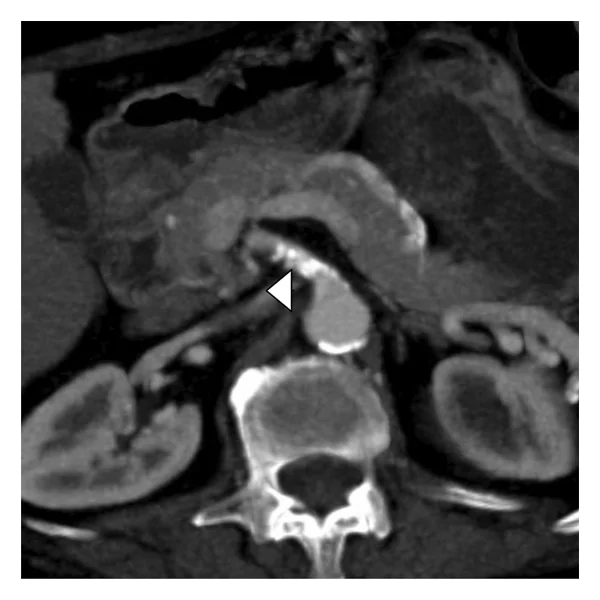
Axial contrast–enhanced abdominal CT image demonstrating calcific plaques located at the origin of the SMA. (Arrowhead) The calcifications appear as bright, dense areas, indicating the presence of atherosclerotic plaques at the point where the SMA originates from the abdominal aorta. This calcific buildup can contribute to narrowing of the artery and reduce blood flow to the intestines.

**FIGURE 2:**
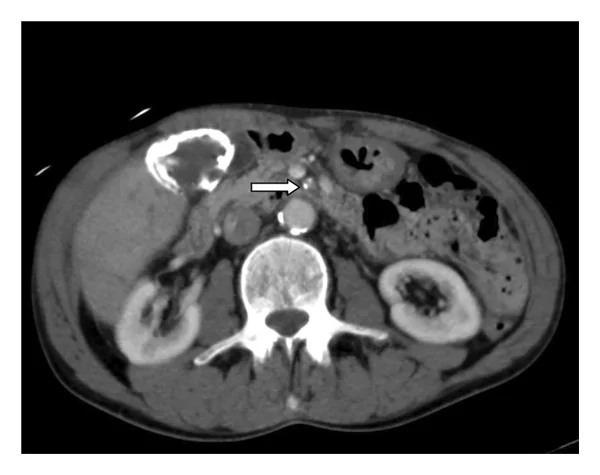
Contrast‐enhanced axial abdomen CT revealing narrowing of the middle segment of the SMA due to the presence of mixed plaque. (Arrow) The mixed plaque, containing both calcified and soft components, results in a reduction of the vessel lumen, potentially leading to compromised blood supply to the bowel, which could cause symptoms of ischemia.

**FIGURE 3:**
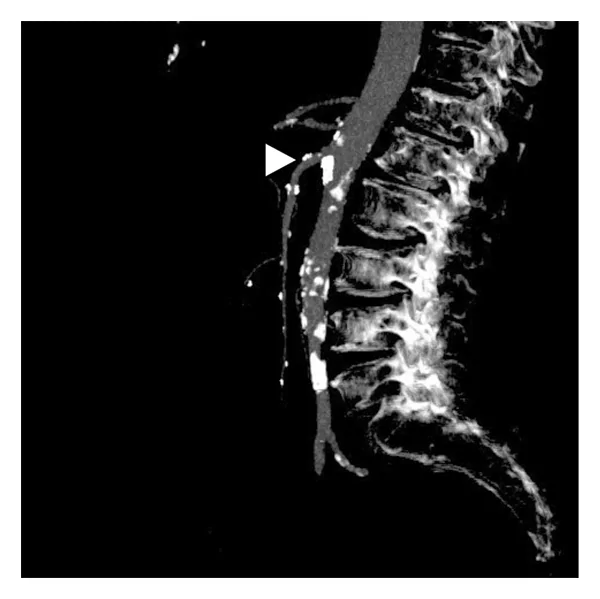
Sagittal maximum intensity projection (MIP) reformats of the abdominal CT angiography display calcific plaques in both the proximal segment of the SMA (arrowhead) and the abdominal aorta. These calcifications appear as bright spots, with the sagittal view providing a longitudinal section that highlights the extent of plaque deposition along the vessel walls.

In our evaluation of plaque distribution in the SMA, we found that plaques were present in 29.4% (38 patients) of the proximal segment (Figure 1), 14.7% (19 patients) of the middle segment (Figure 2), 4.6% (6 patients) of the distal segment, 22.4% (29 patients) of the proximal–middle segment, and 14.7% (19 patients) of the middle–distal segments. Diffuse SMA involvement was observed in 18 patients (13.9%) (Table 1). Overall, plaques were most commonly located in the proximal and transitional segments of the SMA. Most of these plaques were located in the proximal and middle SMA, and all patients exhibited abdominal aortic atherosclerosis (Figure 3).

Atherosclerosis of the celiac trunk was observed in 54 patients (41.8%), and atherosclerosis of the splenic artery was observed in 16 patients (12.4%). Atherosclerotic plaques were observed in the inferior mesenteric artery (IMA), which showed the least atherosclerotic involvement among the abdominal arteries studied. Plaques were observed in 35 patients (27.1%). Of these, 20.2% were calcific, 5.4% were mixed, and 1.6% were soft‐type plaques (Table 2).

**TABLE 2:** Distribution of atherosclerotic involvement in visceral and renal arteries.

Arterial involvement	*n*	%
Celiac trunk	54	41.8
Splenic artery	16	12.4
Inferior mesenteric artery (IMA)	35	27.1
Renal artery involvement	93	72.1

**Renal artery involvement patterns**		
**Pattern**	***n***	**%**

Bilateral calcified plaques	52	40.3
Bilateral mixed plaques	11	8.5
Right renal artery plaques	20	15.5
Left renal artery plaques	10	7.8
No renal artery involvement	35	27.9
Total	129	100

Abbreviation: IMA: inferior mesenteric artery.

Calcific plaques were found in 11 patients (8.5%), soft plaques in 7 patients (5.4%), and mixed truncal plaques in 35 patients (27.9%). Calcific plaques were found only in the splenic artery in 18 patients (12.4% of patients).

This study also examined the potential coexistence of atherosclerosis in other visceral arteries. Renal artery atherosclerosis was most commonly observed in the visceral arteries.

**FIGURE 4:**
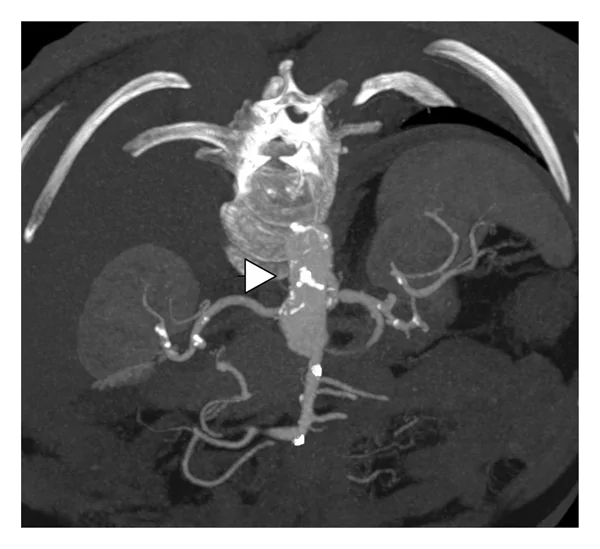
A contrast‐enhanced axial abdomen CT angiography MIP scan exhibiting calcific plaques not only in the abdominal aorta and SMA but also in the bilateral renal arteries. Widespread calcifications suggest extensive atherosclerosis affecting multiple major abdominal vessels, which can lead to reduced blood flow to vital organs, such as the kidneys and intestines.

Renal artery atherosclerosis was the most frequently observed concurrent involvement of the visceral arteries. Among the patients, 93 (72.1%) exhibited renal artery involvement and 36 (27.9%) did not. Bilateral renal artery atherosclerosis was identified in 63 patients (48.8%), with 52 (40.3%) having bilateral calcified plaques (Figure 4) and 11 (8.5%) having bilateral mixed plaques. Unilateral renal artery plaques were found in 30 patients (23.3%), with 20 patients (15.5%) having plaques in the right renal artery and 10 (7.8%) in the left renal artery.

Among the risk factors associated with atherosclerosis, HT, DM, CAD, and hypercholesterolemia were investigated. We had access to the medical records and laboratory tests of only 102 of the 129 patients. HT was detected in 75 patients (73.5%), DM in 52 (49%), and CAD in 45 (44.1%). Low HDL (< 40 mg/dL) was found in 25 patients (25%), high total cholesterol (> 200 mg/dL) in 38 patients (38%), and high LDL (> 130 mg/dL) in 34 patients (34%), as shown in Table 3.

**TABLE 3:** Distribution of serum lipid profile parameters among patients with SMA atherosclerosis.

Lipid parameter	Category	*n*	%	*p* value
Total cholesterol	< 200 mg/dL	62	62.0	> 0.05
	≥ 200 mg/dL	38	38.0	

HDL	≥ 40 mg/dL	74	74.7	> 0.05
	< 40 mg/dL	25	25.3	

LDL	< 60 mg/dL	9	9.0	> 0.05
	60–130 mg/dL	57	57.0	
	> 130 mg/dL	34	34.0	

VLDL	< 40 mg/dL	85	85.0	> 0.05
	≥ 40 mg/dL	15	15.0	

Abbreviations: HDL: high‐density lipoprotein; LDL: low‐density lipoprotein; VLDL: very low‐density lipoprotein.

Pearson’s chi‐square test was used to assess the relationship between key cardiovascular risk factors and both the morphology and anatomical distribution of plaques in the SMA. The analysis revealed no statistically significant link between HT, DM, or CAD and plaque type (calcified, mixed, or soft) (*p* > 0.05 for all comparisons). Likewise, no significant correlation was found between these cardiovascular risk factors and the location of plaques within the SMA (proximal, middle, distal, proximal–middle, middle–distal, or diffuse involvement) (*p* > 0.05 for all analyses). A detailed summary of the statistical results is presented in Table 4.

**TABLE 4:** Association between cardiovascular risk factors and SMA plaque characteristics using Pearson’s chi‐square test.

Variable	Outcome	*χ* ^2^	df	*p* value
Hypertension	Plaque type	2.14	2	0.343
Diabetes mellitus	Plaque type	1.87	2	0.392
Coronary artery disease	Plaque type	3.02	2	0.221
Hypertension	Plaque location	5.91	5	0.315
Diabetes mellitus	Plaque location	4.83	5	0.437
Coronary artery disease	Plaque location	6.27	5	0.281

Abbreviation: df: degrees of freedom.

Furthermore, none of the patients in this study exhibited definitive CT findings indicative of acute mesenteric ischemia at the time of imaging.

## 3. Discussion

Atherosclerosis is a systemic disease with various clinical presentations, commonly found in major bifurcation regions and areas of turbulent flow [14]. It is a major cause of aneurysm formation and occlusive vascular disease, particularly in elderly patients. Hyperlipidemia, HT, smoking, diabetes, and systemic diseases are predisposing risk factors that affect the arteries and contribute to the development of the disease. HT and DM lead to endothelial dysfunction [15].

Recent studies have underscored the significance of chronic inflammation, oxidative stress, and endothelial shear stress in the onset and development of plaques [16]. Inflammatory agents such as interleukin‐6 (IL‐6), tumor necrosis factor‐alpha (TNF‐α), and C‐reactive protein (CRP) are closely linked to plaque instability and cardiovascular incidents [17, 18]. Furthermore, advancements in molecular imaging have revealed that vulnerable plaques are not only defined by lipid‐rich cores but also exhibit heightened macrophage activity and neovascularization.

Atherosclerotic plaques are unstable, with soft fatty plaques posing a higher risk of rupture than calcific plaques, which are more stable. Plaque rupture can lead to vessel occlusion, infarction, and necrosis if the collateral vessels are insufficient to supply the organ. Larger branch thromboses result in ischemia and infarction.

Acute mesenteric ischemia is a life‐threatening vascular emergency with a mortality rate of 50%–80% [19] that typically arises from atherosclerotic stenosis or thromboembolism of the proximal part of the SMA. SMA thrombosis usually results in near‐total occlusion of the middle colic artery. Without adequate collateral flow from the IMA and celiac trunk, blood flow to the jejunum and ileum is reduced, leading to ischemia.

Recent radiological research has highlighted that mixed plaques may fall into the intermediate‐risk category, as they exhibit characteristics of both stability and vulnerability. These plaques are increasingly acknowledged as important clinical indicators of potential adverse vascular events [20]. Furthermore, quantitative plaque analysis using CT, which includes Hounsfield unit characterization and evaluation of plaque burden, has enhanced the ability to stratify the risk in both coronary and noncoronary vascular regions.

Approximately 3%–4% of arterial emboli affect the SMA [21]. In a study of 102 cases of SMA occlusion by Schnee et al., 33% of SMA occlusions were caused by emboli, 26% by thrombus, and 22% by nonocclusive causes. In addition, vasoconstriction may accompany thrombus or embolism, further increasing ischemia [22].

Recent studies based on population data indicate that acute mesenteric ischemia represents approximately 0.1%–0.2% of all hospital admissions; however, it is associated with a notably high mortality rate, especially if the diagnosis is delayed [23].

Identifying the condition within the first 6 h has been proven to significantly lower mortality rates, highlighting the importance of prompt imaging and treatment. In selected patients, endovascular techniques, such as percutaneous thrombectomy and stenting, are increasingly favored over open surgery because of their reduced morbidity and better outcomes [24].

Intestinal ischemia presents with a variety of clinical and radiological findings, ranging from transient ischemic attacks to necrosis. Atherosclerosis is the primary cause, but other factors include trauma, vasculitis, radiation, chemotherapy, intra‐abdominal inflammation, and malignancy [25]. Abdominal CT is a rapid, noninvasive diagnostic method that reveals both the etiology and complications. Additionally, MDCT is highly sensitive and specific, allowing for the evaluation of the mesentery, bowel wall, and vascular structures. CT findings of mesenteric ischemia include bowel wall thickening, mesenteric or portal vein thrombosis, intramural air, and bowel wall enhancement [16].

The Society for Vascular Surgery (SVS, 2021) has recently issued guidelines suggesting that contrast‐enhanced CTA should be the primary imaging technique for diagnosing suspected mesenteric ischemia, with sensitivity and specificity rates of over 90%. The use of dual‐energy CT and perfusion CT methods has further enhanced diagnostic precision by enabling the evaluation of bowel perfusion and detection of early ischemic changes before they become irreversible [26].

Patients with chronic mesenteric ischemia present with recurrent postprandial abdominal pain, unlike those with acute cases. Chronic mesenteric ischemia is usually caused by atherosclerosis, which leads to lumen narrowing and reduced blood flow. Over time, collateral vessels may form, maintaining bowel perfusion in some patients. CT can detect calcific plaques and assess vascularity in asymptomatic older adults [27].

In a histological analysis by Schnee et al. of 21 postmortem elderly cases, 62% had advanced‐stage atherosclerosis of the SMA [28]. MDCT is an effective noninvasive tool for assessing plaque morphology and density, allowing for a more detailed examination and reconstruction.

MDCT allows for clear visualization of the marginal branches of the SMA up to the vasa recta, whereas MPR images and 3D CT angiographic reconstructions offer a comprehensive evaluation of vascular anatomy, atherosclerotic plaques, collateral circulation, and ischemic complications [27].

Recent technological advancements, including photon‐counting CT and AI‐based image analysis, have greatly enhanced the ability to detect and characterize atherosclerotic plaques. AI‐driven algorithms can automatically assess plaque burden, determine plaque composition, and reliably predict stenosis severity with high accuracy [29]. These technological innovations may improve the early detection and risk evaluation of visceral artery atherosclerosis.

MDCT outperforms single‐detector CT, particularly in CTA. The use of water as an oral contrast agent improves the visualization of the bowel wall and mesenteric vasculature. Currently, multislice CT with 3D reformatting is used in patients with suspected acute or chronic mesenteric ischemia. This approach reduces the need for additional imaging studies, such as Doppler ultrasound or angiography. Further research is required to optimize the use of MDCT in these clinical conditions.

Moreover, in most clinical environments, contrast‐enhanced CTA has become the preferred diagnostic method, largely replacing traditional catheter angiography, which is now primarily used for therapeutic procedures [30].

**FIGURE 5:**
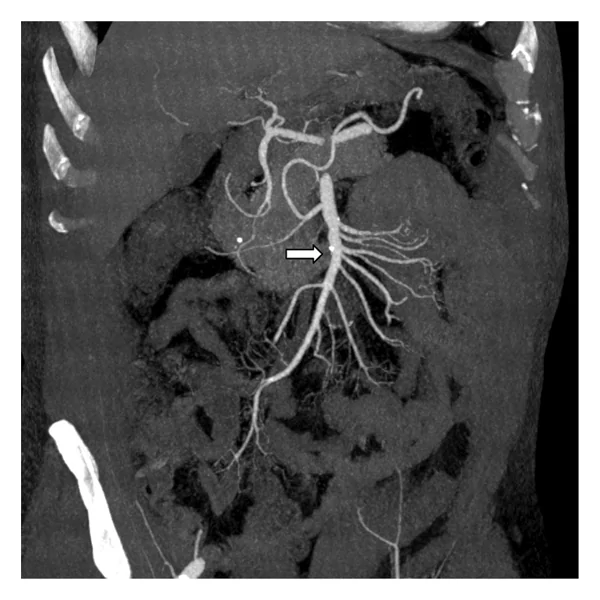
The coronal MIP view from a contrast‐enhanced abdominal CT angiography highlighting the calcific plaques located in the middle section of the SMA. This projection provides a clear view of plaque distribution along the artery, which can lead to stenosis and impair blood flow, potentially resulting in mesenteric ischemia.

Advances in MDCT technology have greatly improved the diagnosis and assessment of atherosclerosis, including the identification of plaque type, structure, and location, as well as the treatment of complications. The visualization of atherosclerotic plaques has become quicker and more precise with modern MDCT technology, enabling high‐resolution thin‐slice scans that create detailed 3D images (Figure 5).

Atherosclerosis was predominantly found in the proximal and transitional sections of the SMA, particularly in the proximal–middle and middle–distal areas. This distribution pattern may indicate the impact of local hemodynamic stress and turbulent flow in arterial transition zones, which are recognized as factors contributing to plaque formation.

In terms of plaque type Calcific, mixed, and soft plaques were observed with decreasing frequencies. Therefore, the absence of calcific plaque in the SMA on noncontrast CT cannot be used to rule out atherosclerosis. The incidence of SMA disease was 0.86%, and it was strongly associated with aorta and renal artery atherosclerosis.

Our results align with recent extensive imaging research, indicating that visceral artery atherosclerosis seldom occurs independently and is closely linked to systemic vascular disease, especially affecting the aorta and renal arteries [31]. Furthermore, the predominance of proximal SMA involvement found in our study supports hemodynamic theories proposing that increased shear stress and turbulence at the vessel’s origin are significant factors in plaque development.

## 4. Conclusion

In conclusion, this study highlights the prevalence and characteristics of atherosclerotic changes in the SMA using MDCT. Among 15,000 scans reviewed, 129 cases of SMA atherosclerosis were identified, with calcific plaques being the most common. These findings emphasize the association between SMA atherosclerosis and common risk factors such as HT, DM, and CAD. Additionally, this study highlights the co‐occurrence of atherosclerosis in other visceral arteries. MDCT is an effective diagnostic tool for early detection, potentially improving patient outcomes through timely intervention and targeted treatment strategies. This study provides valuable insights into the clinical significance of SMA atherosclerosis.

### 4.1. Limitation and Future Implications

The primary limitation of this study is its retrospective nature, which relies on patient records, potentially introducing selection bias and incomplete data. Additionally, the sample size of 129 patients, although representative, may not fully capture the prevalence of SMA atherosclerosis in the general population. Future research should focus on larger prospective studies to validate these findings and investigate the long‐term clinical outcomes of SMA atherosclerosis. Furthermore, incorporating advanced imaging techniques and exploring the role of emerging risk factors, such as genetic predisposition and lifestyle modification, will further enhance diagnostic accuracy and treatment strategies.

## Funding

The author declares that no funds, grants, or other support were received during the preparation of this manuscript.

## Consent

The requirement for informed consent was waived by the institutional review board due to the retrospective nature of the study and the use of anonymized patient data.

## Conflicts of Interest

The author declares no conflicts of interest.

## Data Availability

The datasets generated and analyzed during the current study are available from the corresponding author upon reasonable request.
